# Complex object motion represented by context‐dependent correlated activity of visual interneurones

**DOI:** 10.14814/phy2.13355

**Published:** 2017-07-17

**Authors:** Paul C. Dick, Nicole L. Michel, John R. Gray

**Affiliations:** ^1^ Department of Biology University of Saskatchewan Saskatoon Saskatchewan Canada; ^2^ National Audubon Society San Francisco California

**Keywords:** Locust, motion detection, multineuronal activity, vision

## Abstract

Accurate and adaptive encoding of complex, dynamic visual information is critical for the survival of many animals. Studies across a range of taxa have investigated behavioral and neuronal responses to objects that represent a threat, such as a looming object approaching along a direct collision course. By investigating neural mechanisms of avoidance behaviors through recording multineuronal activity, it is possible to better understand how complex visual information is represented in circuits that ultimately drive behaviors. We used multichannel electrodes to record from the well‐studied locust nervous system to explore how object motion is reflected in activity of correlated neural activity. We presented locusts (*Locusta migratoria*) with objects that moved along one of 11 unique trajectories and recorded from descending interneurons within the ventral nerve cord. Spike sorting resulted in 405 discriminated units across 20 locusts and we found that 75% of the units responded to some form of object motion. Dimensionality reduction through principal component (PCA) and dynamic factor (DFA) analyses revealed population vector responses within individuals and common firing trends across the pool of discriminated units, respectively. Population vector composition (PCA) varied with the stimulus and common trends (DFA) showed unique tuning related to changes in the visual size and trajectory of the object through time. These findings demonstrate that this well‐described collision detection system is more complex than previously envisioned and will drive future experiments to explore fundamental principles of how visual information is processed through context‐dependent dynamic ensembles of neurons to initiate and control complex behavior.

## Introduction

Encoding complex visual information is critical for the survival of many animal species that rely on robust, coordinated and appropriately timed avoidance behaviors. Salient visual cues are important across many taxonomic groups, including humans (Vallis and McFadyen 2003, 2005 Gray and Regan 2006; Poljac et al. 2006), other primates (Maier et al. 2004), gerbils (Ellard 2004), birds (Sun and Frost 1998; Cao et al. 2004), frogs (Yamamoto et al. 2003), fish (Gallagher and Northmore 2006; Preuss et al. 2006), crabs (Oliva et al. 2007; Oliva and Tomsic 2012), and insects (Robertson and Johnson 1993a; Jablonski and Strausfeld 2001; Verspui and Gray 2009; Yamawaki and Toh 2009). Natural animal behavior operates in closed loop. Behaviors evoked by spatiotemporal stimulus properties result in changes in the stimulus that must be encoded and decoded appropriately to allow subsequent behaviors to be adaptive. For example, objects approaching along a direct collision course reflect movement of the animal through space as well as motion of conspecifics or predators. However, even within a single modality, natural stimuli consist of ranges of properties that are encoded by receptors and downstream networks as well as range fractionation at multiple processing levels. Therefore, a more complete understanding of how vision controls even “simple” behaviors, such as avoidance, requires study of parallel visual pathways that encode changing spatiotemporal stimulus properties.

Multineuronal recordings and analysis allow researchers to investigate how populations of neurons encode and process sensory information (see Ruther and Paul 2015 for a review). Populations are often characterized as ensemble of neurons in which the activity of each unit combines in some functional manner and population responses can be reflected in parallel recordings using multichannel electrodes. As such, emergent firing of neural populations can provide a mechanism to disambiguate variation in unit firing that results from presentation of complex natural stimuli. Without emergent population coding, individual unit activity could produce an intractable barrage of firing patterns that would not be effectively decoded by downstream elements involved in production of coordinated behaviors. In this context, population coding is important for processing visual information used for motion detection of objects or an individual's movement through space. In the medial temporal area of the extrastriate visual cortex in Rhesus monkeys, population responses predict effects of spatial frequency and contrast on target speed estimations (Priebe and Lisberger 2004). Population responses in human visual cortex can predict the identity of an image and prediction accuracy improves with the number of units (Quiroga et al. 2007). In *Drosophila* ellipsoid body, ensembles encode the fly's azimuth relative to its environment, which is maintained through persistent activity in the absence of stimuli (Seelig and Jayaraman 2015). These studies demonstrate that population coding is an important property of visual detection and perception. Studying populations of visual sensory neurons in tractable systems with well defined, visually guided behaviors will allow future experiments to address fundamental questions of how animals use complex cues to drive adaptive responses.

**Figure 1:**
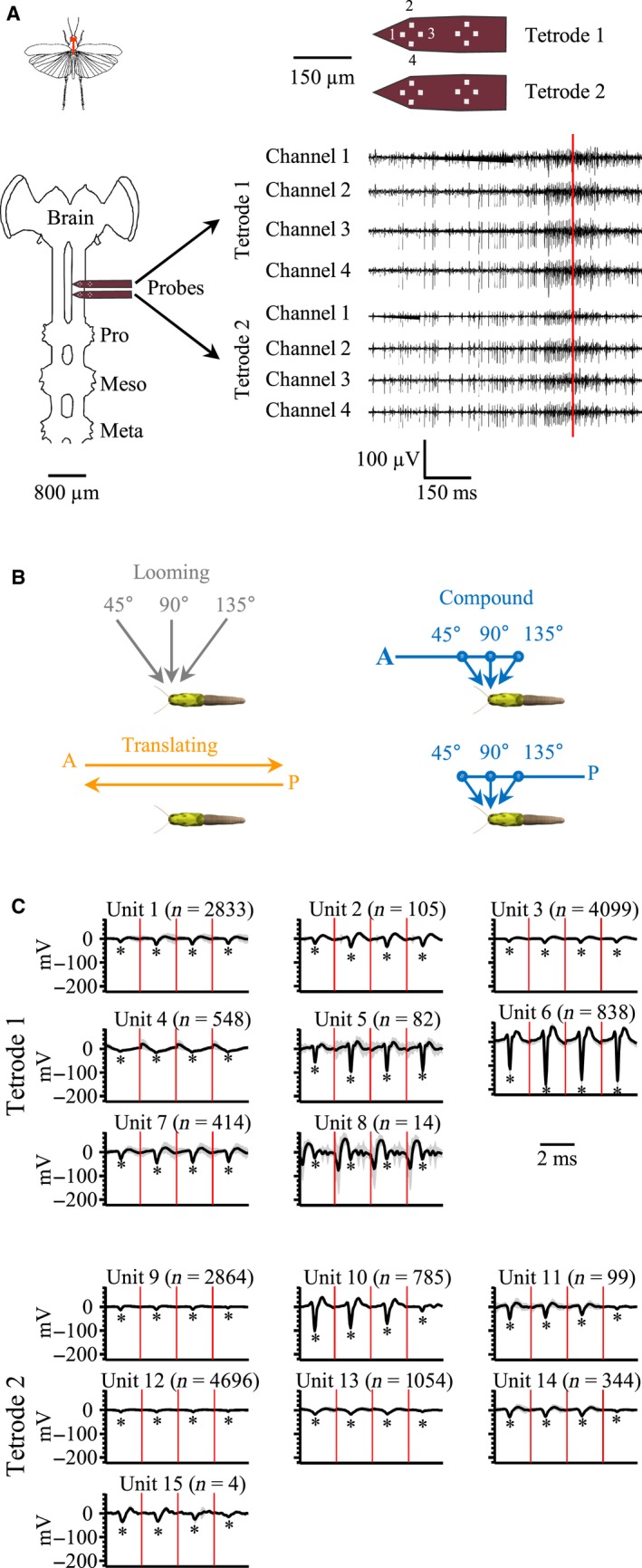
Experimental setup and visual stimuli. (A) The diagram of the locust shows the relative position of the central nervous system (CNS) in red. The expanded (ventral) view of the CNS below shows the relative positions of the brain and the prothoracic (Pro), mesothoracic (Meso), and metathoracic (Meta) ganglia in the thorax. One set of distal tetrodes on each shank of a silicon multichannel probe were inserted into the ventral connective anterior to the prothoracic ganglion. Upper right shows an expanded view of the probes indicating the recording sites on one tetrode corresponding to data channels on the top probe (Tetrode 1). We also used corresponding recording sites on Tetrode 2 (see materials and Methods). Raw neural recordings (bottom right) were taken from a single locust presented with a looming disk and shown for each of eight channels across the two tetrodes. The red vertical line indicates the projected time of collision. (B) Eleven different stimuli were presented as either directly looming (gray), translating from anterior or poster (orange) or compound trajectories with transitions (small filled circles) from anterior or posterior translation to a looming trajectory (blue). Each random sequence of stimuli was bracketed by a straight loom from 90° to rule out the potential effects of the duration of the experiment on DCMD responses (see Materials and Methods). (C) Stability of waveform shapes throughout the experiment duration. Average waveforms (black lines) and standard deviation (gray shade) from discriminated units. Data from each tetrode recording from one animal across 13 stimulus presentations. Each panel is divided into four sections of 2.048 msec, delimited by red vertical lines, representing time across each recording site. *n* = number of waveforms for each unit. Asterisks indicate the valley of the detected waveform.

The migratory locust (*Locusta migratoria*) is an excellent system for studying visually guided control of complex behaviors as there is a wealth of knowledge regarding robust behavioral and neural responses to approaching objects. Jumping responses to a looming object (Fotowat and Gabbiani 2007; Fotowat et al. 2011) follow a precisely timed sequence that relates to properties of motion‐sensitive neurons. Similarly, intentional flight steering in response to a loom involves production of forewing asymmetries and steering torques (Robertson and Johnson 1993a,b; Chan and Gabbiani 2013) that are driven by adjustments in the timing of wing muscles (McMillan et al. 2013). One motion‐sensitive pathway in locusts includes the Lobula Giant Movement Detector (LGMD) and it postsynaptic partner, the Descending Contralateral Movement Detector (DCMD), which receives retinotopic inputs from the compound eyes (O'Shea 1976) and coveys motion information to motor centers that control the legs and wings (Burrows and Rowell 1973; Simmons 1980; Pearson et al. 1985; Boyan 1989). While there is much information on putative biophysical mechanisms that underlie LGMD computation of a looming object's visual properties (Rind and Bramwell 1996; Gabbiani et al. 2004; Jones and Gabbiani 2010; Fotowat et al. 2011), relatively few studies have attempted to directly relate the activity of this pathway to flight steering (Santer et al. 2006). Given the variability of flight steering behaviors in locusts able to maneuver within six degrees of freedom (Chan and Gabbiani 2013; McMillan et al. 2013) and the ability of the LGMD/DCMD pathway to respond to complex object motion (Guest and Gray 2006; McMillan and Gray 2012; Dick and Gray 2014) against complex visual backgrounds (Yakubowski et al. 2016), it is likely that other motion‐sensitive descending interneurons (Griss and Rowell 1986; Rowell and Reichert 1986; Gray et al. 2010) play a role in initiating and coordinating this complex behavior. A better understanding of the neural mechanisms of collision detection and avoidance during flight requires simultaneous recordings of multiple descending interneurons when presented with complex object motion. As a first step in testing the hypothesis that object visual motion is coded in parallel descending neural activity, we recorded from the ventral connective of *L. migratoria* using multichannel electrodes while presenting a variety of visual stimuli that included direct approaches (looming) from different regions of the visual field, translation across the visual field, and transitions from translation to looming (Fig. 1). Using spike sorting, principal component analysis, and dynamic factor analysis, we were able to reduce the dimensionality of single unit firing across a pooled set of neurons and we found that correlated firing of discriminated units reflects stimulus properties of objects moving along simple and compound trajectories.

## Materials and Methods

### Animals

Adult male locusts (*Locusta migratoria*) were obtained from a crowded colony maintained at the University of Saskatchewan in the Department of Biology, kept at 25–28°C with a 12 h light‐dark cycle. Locusts were selected at no less than 3 weeks past the imaginal molt, and experiments were carried out at room temperature (~25°C).

### Preparation

Following restraint of the wings and removal of the legs, a rigid tether was attached to the ventral surface of the thorax with melted beeswax. A square of anterior thoracic cuticle was removed using a sapphire blade to expose the paired connectives of the ventral nerve cord between the subesophageal and prothoracic ganglia. Following removal of air sacs and fat body, the translucent protective sheath surrounding the left connective was carefully cut using the sapphire blade and removed using fine forceps. The tissue was bathed in a drop of locust saline (in mmol: 147 NaCl, 10 KCl, 4 CaCl2, 3 NaOH, 10 HEPES, pH 7.2) and the preparation was transferred to the recording stage. Following insertion of a silver ground wire in to the abdomen, a multichannel electrode with 2 × 2 tetrode arrays of 16 channels (NeuroNexus Technologies, MI, USA) was mounted onto a Narishige MM‐3 manipulator (Narshige, NY, USA) and carefully inserted in to the desheathed connective. The tetrode array was maneuvered until a high signal:noise recording was achieved on at least four recording sites. The two shanks of the electrode array were arranged parallel to the long axis of the nerve chord such that one of the shanks was anterior and one was posterior. Given the depth of the connective and the spacing of the tetrode on each shank, we used the distal most tetrodes on either shank (Fig. 1A). Therefore, each recording used eight recording sites within an anterior and posterior tetrode array. The entire preparation was then rotated so that the locust was oriented dorsal side up perpendicular to and 10 cm away from the apex of a rear projection dome screen aligned to the center of the right eye. The preparation was left for 15 min in front of a projected white visual field (background luminance = 430 cd/m^2^) to allow acclimation to the experimental setup. In this configuration, we designated azimuthal coordinates such that 0° was in front of the locust, 90° was perpendicular to the center of the eye (the dome apex), and 180° was directly behind the locust.

### Visual stimuli

The procedure for generation of visual stimuli was similar to that used by (Guest and Gray 2006). Vision Egg software (Straw 2008) running on a Python programming platform rendered visual stimuli as 1024 × 1024 pixel portable network graphics (png) files. Individual projected pixels were ~0.7 mm, subtending a visual angle of ~0.4°, which is below the acceptance angle of ommatidia of the locust compound eye (Horridge 1978). Stimuli were projected onto a specialized rear projection dome screen with an InFocus DepthQ LCD data projector at 85 frames/s, which is above the flicker fusion frequency of the locust eye (Miall 1978). Correction factors embedded in the Vision Egg code accounted for distortion due to projection onto the curved surface. A 1.2‐msec TTL pulse included in each video frame and the vertical refresh synchronization pulse (vsync) from the video card (NVIDIA GeForce4 Ti4200 128 MB) were used to align physiological recordings with the stimuli. The last TTL pulse was used to determine the final frame of the presentation, indicating the disappearance of the object from the screen. The corresponding vsync pulse thus determined the start of frame rendering. All visual stimuli involved the movement of a 7‐cm‐diameter black disk traveling at 3 m/sec along predesigned trajectories. The luminance values and Michelson contrast ratio (0.48) have been used previously (Guest and Gray 2006). To test neural responses to complex visual motion, we presented each of 20 locusts with 11 different trajectories based on a subset from (McMillan and Gray 2012): straight looming along 45°, 90°, or 135° azimuth, translation parallel to the locust longitudinal body axis at 80 cm from the eye and three compound trajectories that began as translating and transitioned to looming at 45°, 90°, or 135° azimuth (Fig. 1B). All trajectories with translational components were repeated for both directions of motion, toward the posterior or toward the anterior. We used the following naming conventions to simplify descriptions of the stimuli: approaches with only a looming component were designated by the direction of approach (45, 90, 135), movement with only a translational component were designated by the direction of motion (A  =  motion from anterior to posterior, P  =  motion from posterior to anterior), and compound trajectories were designated based on the direction of initial translation and subsequent direction of the looming component (e.g., A45 =  translation from anterior to posterior transitioning to looming at 45° in the azimuthal plane). For each locust, the sequence of stimuli was presented in a different random sequence and each sequence was bracketed by a 90° loom presented at both the beginning and end to rule out the potential effects of the duration of the experiment on DCMD responses. Therefore, in total, each locust was presented with 13 stimuli. However, for analysis of looms from 90° we only used the single approach that was within the randomized sequence. Therefore 11 different trajectories were used for analysis. To prevent confounding effects of neural habituation, the interval between each presentation was at least 3 min.

### Data acquisition, spike detection, and unit discrimination

For each stimulus presentation, we recorded and stored neuronal activity data from eight channels, pulses from each frame of the stimulus, and vsync pulses. Neural activity was amplified with an RA16PA Medusa preamp (Tucker‐Davis Technologies, Alachua, FL) and sampled at 25 kHz, whereas vsync and frame pulses were amplified with an RA8GA Loggerhead preamplifier. An RX5 Pentusa Base Station with Butterworth filter settings of 100 Hz (high pass) and 5 kHz (low pass) was used to store the data to disc. For spike detection and waveform sorting, we imported recorded data into Offline Sorter v. 4.1.0 (Plexon Inc. Dallas, TX). Neural data channels were arranged into a tetrode configuration based on channel mapping during initial data acquisition. Individual data files contained data from one stimulus presentation to one animal, which were presented in a different random order for each locust. These files were combined into one data file per animal that contained all stimulus types reordered such that they were in the same sequence for each locust. Thus, spike detection and waveform sorting were performed on a single data file for each animal. Data from the stimulus and vsync pulses were imported separately into Offline Sorter and combined in the same order for each locust. Stimulus and vsync pulses generated event times used to align the data alignment with the stimulus for spike train analysis.

The threshold for spike detection of physiological data was set at three standard deviations from the mean voltage on each imported continuous data channel. We sorted detected spikes using a semiautomatic process that initially used the T‐distribution E‐M algorithm from Offline Sorter, which is a variant of the E‐M algorithm (Shoham et al. 2003). Pilot manual sorting of waveforms from a single stimulus presentation to one animal revealed approximately 20 discriminated units. Given that the T‐Distribution E‐M algorithm agglomerates clusters through an iterative process, we set the number of initial “seed” clusters (units) to 35 to avoid under‐sampling the number of units. Following an initial automatic sort, units were further discriminated manually based on waveform shape and amplitude (Fig. 1C). We set a minimum inter‐spike interval of 1 ms to avoid double counting of overlapping waveforms within individual discriminated units. To determine that recordings were stable throughout the duration of stimulus sequences (approximately 40 min), we calculated the average and standard deviation from the entire dataset for each locust.

### Spike train analysis

Spike times for discriminated units were exported into Neuroexplorer spike train analysis software (Nex Technologies, Plexon Inc., Dallas, TX) and aligned to either the projected time of collision (TOC) for trajectories with looming components or the time the object crossed 90° azimuth (T90) for translation‐only trajectories.

Given the orientation of our tetrode arrays (see above) it was possible that the same unit could have been recorded across both tetrodes. Therefore, we used cross correlograms (bin width = 1 msec) to determine if units were represented twice in each animal. The range of distances between recording sites on the probes was 114–183 *μ*m. For single units to be represented twice within a 1 msec time window across these recording site distances, the conduction velocity of the axons would need to be 0.11–0.18 m/sec, which is slower than known values from descending motion‐sensitive neurons. (Gray et al. 2010) reported conduction velocities of 2.6 and 2.0 m/sec for DCMD and LDCMD, respectively. At a conduction velocity of 2.6 m/sec (e.g., DCMD) a spike would take 0.09 msec to spread across the farthest distance between recoding sites and for a conduction velocity of 2.0 m/sec (e.g., LDCMD) it would take 0.06 msec to spread across the shortest distance. Therefore, a time window of 1 msec is sufficiently long enough to resolve the same spike recorded by each tetrode. Using this rationale, we detected a pair of duplicate units from 1 locust and triplicate units from another. Therefore, from the 408 discriminated units across all locusts, we determined that 405 were distinct (Table 1).

**Table 1:** Summary statistics for sorted spikes across each tetrode for each animal

Locust	Number of spikes			Total discriminated units	MANOVA	
	Tetrode 1	Tetrode 2	Total		Tetrode 1	Tetrode 2
L01	1317	1508	2825	15	*F*_16,2614_ = 5.1, *P* ≪ 0.001	*F*_12,3000_ = 15.5, *P* ≪ 0.001
L02	26,576	13,014	39,590	28	*F*_30,52788_ = 3.7, *P* ≪ 0.001	*F*_22,26002_ = 3.1, *P* ≪ 0.001
L03	20,093	13,521	33,614	30	*F*_28,40152_ = 6.3, *P* ≪ 0.001	*F*_30,27008_ = 4.1, *P* ≪ 0.001
L04	8104	7906	16,010	23	*F*_14,16190_ = 3.1, *P *≪ 0.001	*F*_28,15780_ = 5.7, *P *≪ 0.001
L05	10,948	12,178	23,126	30	*F*_18,21874_ = 9.3, *P* ≪ 0.001	*F*_38,24314_ = 33.7, *P *≪ 0.001
L06	12,368	13,579	25,947	28	*F*_34,24698_ = 14.8, *P *≪ 0.001	*F*_18,27136_ = 4.0, *P *≪ 0.001
L07	12,561	10,762	23,323	25	*F*_26,25092_ = 6.5, *P *≪ 0.001	*F*_20,21500_ = 7.9, *P *≪ 0.001
L08	17,110	14,329	31,439	15	*F*_14,24214_ = 55.6, *P *≪ 0.001	*F*_12,28640_ = 14.7, *P* ≪ 0.001
L09	8106	10,091	18,197	10	*F*_10,16198_ = 4.6, *P* ≪ 0.001	*F*_6,20172_ = 10.9, *P* ≪ 0.001
L10	10,549	10,950	21,499	22	*F*_18,21076_ = 51.0, *P* ≪ 0.001	*F*_22,21874_ = 4.9, *P* ≪ 0.001
L11	14,967	11,954	26,921	24	*F*_24,29906_ = 2.2, *P* < 0.001	*F*_20,23884_ = 5.7, *P* ≪ 0.001
L12	10,841	9,054	19,895	15	*F*_10,21668_ = 2.3, *P* = 0.011	*F*_16,18088_ = 3.8, *P *≪ 0.001
L13	13,638	12,885	26,523	20	*F*_12,27260_ 3.2, *P* < 0.001	*F*_24,25742_ = 3.6, *P *≪ 0.001
L14	12,857	13,834	26,691	22	*F*_22,25688_ = 24.5, *P* ≪ 0.001	*F*_18,24832_ = 4.3, *P* ≪ 0.001
L15	11,695	11,569	23,264	18	*F*_12,23374_ = 4.8, *P *≪ 0.001	*F*_20,23114_ = 26.3, *P* ≪ 0.001
L16	8933	9846	18,779	15	*F*_14,17848_ = 4.5, *P *≪ 0.001	*F*_12,19676_ = 3.2, *P* < 0.001
L17	7779	11,073	18,852	14	*F*_14,15540_ = 7.5, *P *≪ 0.001	*F*_10,22132_ = 12.3, *P *≪ 0.001
L18	14,989	12,519	27,508	19	*F*_18,29956_ = 2.0, *P* = 0.009	*F*_16,25018_ = 5.8, *P *≪ 0.001
L19	8084	11,245	19,329	12	*F*_8,16156_ = 6.1, *P *≪ 0.001	*F*_12,22474_ = 8.9, *P *≪ 0.001
L20	9411	10,601	20,012	20	*F*_16,18802_ = 4.5, *P *≪ 0.001	*F*_24,21174_ = 2.7, *P* < 0.001
Total	240,926	222,418	463,344	405		
Mean	12,046	11,121	23,167	20		
Median	11,322	11,407	23,195	20		
S.D.	5252	2812	7530	6		
Min	1317	1508	2825	10		
Max	26,576	14,329	39,590	30		

**Figure 2:**
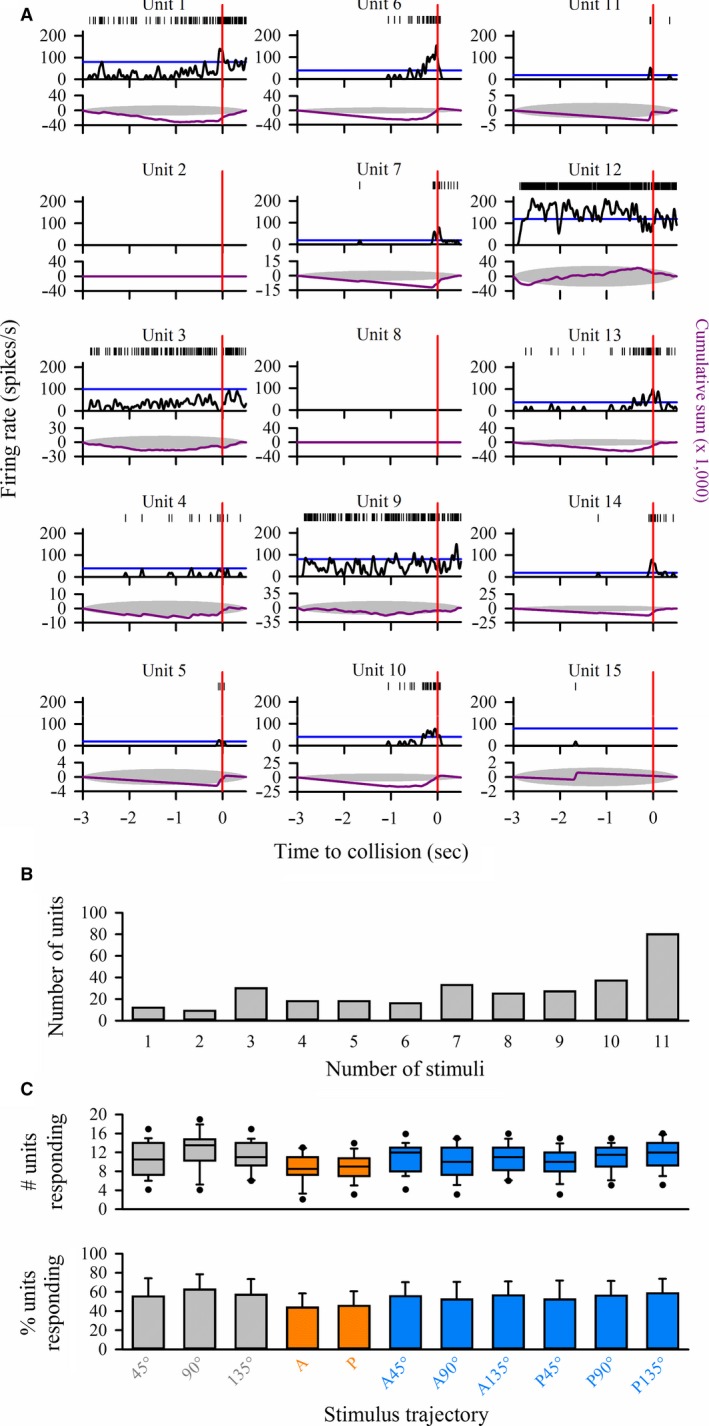
(A) Identification of responding units. Representative plots of 15 discriminated units from one locust responding to a disk looming from 90°. Time of projected collision is indicated by the red vertical line. Upper plots show rasters of spike times. Middle plots show peristimulus time histograms using a 1 msec bin and 50 msec Gaussian smoothing filter. The blue horizontal line is the 95% confidence interval of the histogram. Bottom plots show the cumulative sum (purple line) against a 99% confidence interval ellipse (gray shade). Units 2 and 8 did not generate any spikes and unit 15 showed no significant change in firing rate, indicating that these three units did not respond to the stimulus. The remaining units showed some form of response, as indicated by the histogram touching or passing the 95% confidence interval and the cumulative sum touching or extending outside the 99% confidence ellipse. (B) Frequency histogram showing the distribution of units responding to one or more of the 11 stimuli within a randomized sequence. (C) Data from all locusts (*n* = 20) plotting the median number of units (upper panel) and mean percent of all discriminated units (lower panel) that responded for each stimulus type. There were no significant differences in the number or percent of units responding to different stimuli. Gray cells, boxes, and bars represent data from purely looming trajectories, orange boxes and bars from translating trajectories, blue boxes and bars from compound trajectories. Boxes represent the median, 25 and 75th percentiles. For the upper panel, whiskers represent the 10th and 90th percentile and symbols represent the 5th and 95th percentiles. For the lower panel, bars represent the mean and error bars represent the positive standard deviation. (see Materials and Methods for details).

To determine if each discriminated unit responded to the stimulus, we generated a peristimulus time histogram (PSTH – 1 msec bin width and 50 msec Gaussian smoothing filter) aligned to TOC or T90. The PSTH included a 95% confidence level as well as a separate plot of a weighted cumulative sum of spike counts tested against an ellipse that represented the 99% confidence level. The cumulative sum is a routine in Neuroexplorer that was calculated with the following algorithm: for bin 1, cs(1) = bc(1)A; for bin 2, cs(2) = bc(1) + bc(2)‐A X 2; for bin 3: cs(3) = bc(1) + bc(2) + bc(3)‐A X 3, etc. The cumulative sum (cs) at each bin count (bc) includes the counts from previous bins as well as the sum of the current bin count minus the average (A) of the entire histogram. A cumulative sum that did not extend outside the 99% confidence level ellipse was considered to have represented a firing rate that showed no significant change resulting from the stimulus, that is, no response, whereas a cumulative sum that extended outside, or touched, the ellipse was considered to have a significant, stimulus evoked, firing rate change (i.e., a response). Figure 2A shows an example from one locust of units that generated a significant response or no response when presented with a disk approaching from 90°.

### Dimensionality reduction and tuning of common trends

To reduce dimensionality of the data and explore putative common responses across units, we first performed a principal component analysis (PCA) on raw spike times of units within each animal using a 50 msec bin. For trajectories with a looming component we used data from *t* = −1.5 sec to TOC whereas for translating trajectories we used spike times from ± 2 sec relative to T90. The number of components extracted for further analysis was determined by selecting the number of components with Eigenvalues > 1 and accounting for at least 70% of the cumulative variance. We then created PSTH plots for the resulting population vectors to determine if they responded to the stimuli. We considered a vector to have responded if the plot of the standardized firing rate crossed a 95% confidence interval.

Slight variations in tetrode positioning precluded unambiguous recordings of the same neurons from each preparation, resulting in an inability to directly compare population vectors across locusts. Therefore, we pooled responding population vectors across all animals for each stimulus type. The pooled population vectors were analyzed using dynamic factor analysis (DFA), a method for identifying underlying common trends shared among time series (Zuur et al. 2003). DFA is a dimension reduction technique that identifies similarities among population trajectories, similar to principal components analysis for time series. DFA models a set of univariate time series as linear combinations of underlying common trends, explanatory variables, and noise:

(1) $$Y_{iwt}=a_{iw}Mt+c_{iw}+\varepsilon t$$

where *Y*_*iwt*_ is the neural response of the *i*th PC axis of the *w*th individual at time *t* (*i *=* *1, …, *n; w *=* *1, …, 20; and *t *=* *−1.50, …, 0); *ai* is the factor loading for the *i*th PC axis of the *w*th individual; *Mt* is the common trend at time *t*;* c*_*iw*_ is a constant level parameter for the *i*th PC axis of the *w*th individual; and ɛ*t* represents the noise. Factor loadings (*ai;* weights relating observed unit responses and fitted values) indicate how representative a given common trend is for each PC axis for each individual.

Population vector data were standardized (mean subtracted from each value and divided by standard deviation) prior to analysis to facilitate interpretation of factor loadings and common trends (Zuur et al. 2003). Dynamic factor analyses were run twice, once with equal error variance for each time series (indicating similar error among PC axes and individuals), and the second with unequal error variances (indicating different error between PC axes and individuals); both models used diagonal covariance matrices (no covariance among time series). The optimal number of common trends was identified through an iterative process starting with *M *=* *1 common trends and continuing to increase the number of common trends until the model fit worsened (Table 2). Model selection was conducted using a combination of Akaike's Information Criterion adjusted for small sample size (AICc), distribution of the residuals, and biological interpretation (Zuur et al. 2003). All analyses were conducted in R 3.1.1 (Team 2014). Dynamic factor analyses were conducted using the *MARSS* package (Holmes et al. 2012).

**Table 2:** Number of common trends, variance structure, number of parameters, log‐likelihood, AIC_c_, and *w*_i_ (Akaike weight) from dynamic factor models for a 90° loom

Number of common trends	Variance structure	Number of parameters	Log‐likelihood	AIC_c_	*w*_i_
**10**	**DU**	**802**	−**3005.9**	**7934.1**	**1.00**
11	DU	869	−2859.5	7988.2	0.00
9	DU	734	−3135.1	8000.4	0.00
8	DU	665	−3292.6	8126.8	0.00
7	DU	595	−3457.7	8272.1	0.00
6	DU	524	−3616.0	8407.1	0.00
5	DU	452	−3770.0	8537.1	0.00
4	DU	379	−3968.6	8759.7	0.00
3	DU	305	−4166.6	8984.2	0.00
10	DE	726	−3739.7	9187.3	0.00
11	DE	793	−3586.7	9251.9	0.00
2	DU	230	−4400.7	9284.5	0.00
9	DE	658	−3890.2	9303.2	0.00
8	DE	589	−4057.7	9456.4	0.00
7	DE	519	−4218.2	9599.0	0.00
6	DE	448	−4373.7	9734.8	0.00
5	DE	376	−4538.2	9891.8	0.00
4	DE	303	−4682.7	10012.0	0.00
3	DE	229	−4844.2	10169.3	0.00
2	DE	154	−5029.8	10377.7	0.00
1	DU	154	−5203.4	10725.0	0.00
1	DE	78	−5576.9	11312.3	0.00

Best model indicated in bold. DU, dynamic factor analysis with unequal variance; DE, dynamic factor analysis with equal variance (see Methods for details).

To determine which units contributed most to the common trends for a direct loom from 90°, we selected population vectors (for DFA) and subsequent units (from PCA) with factor loadings ≥ 0.3 (DiStefano et al. 2009) on the respective analysis. The combined activity of units that are not part of a putative population could be expressed simply as an average firing rate (equal loadings with no weighted correlations), which could inform the possibility that units are firing independently or in a correlated manner (with weighted correlations). Therefore, we examined responses of each constituent unit as well as the mean across units and common trends from DFA.

### Statistics

All data were tested for normality and equal variance to determine whether further testing required parametric or nonparametric statistics. Across all stimulus types, we tested the median number of responding units using a Kruskal–Wallis one‐way analysis of variance on ranks and the mean percent of responding units using a one‐way analysis of variance. Tests were indicated by the *H* or *F* statistic, respectively, with a subscript to indicate the degrees of freedom. Where appropriate, data that passed normality and equal variance were plotted as the mean and positive standard deviation, nonparametric data were plotted as boxes representing the median, 25 and 75th percentiles, with whiskers representing the 10th and 90th percentile, and symbols representing the 5th and 95th percentiles.

## Results

### Unit discrimination

Spike sorting revealed a total of 463,344 spikes from all 20 locusts (median of 23,195 spikes per locust summed across both sets of tetrodes; range = 2825–9590 spikes per tetrode (Table 1). MANOVA analysis on sorted clusters from each combined data file for each locust revealed that clusters were statistically well‐separated in two‐dimensional space. We discriminated a total of 405 units from 20 locusts (median = 20 units/locust, range = 10–30) that accounted for 100% of the detected spikes. Figure 1C shows the sorted spikes from a single locust that represent 15 discriminated units across the two tetrodes, which were stable throughout the stimulus sequences.

### Unit responses

Figure 2A shows responses of units represented in Figure 1C when presented with a disk looming from 90°. Based on our criteria for determining whether units responded or not (see Materials and Methods), we found that 12 of the 15 units generated significant responses.

Figure 2B shows the distribution of the number of units across the entire dataset (*n* = 20 locusts) that responded to 1 or more of 11 stimuli (not including looming stimuli at the beginning and end of the sequence). We found that while units responded to different numbers of stimuli, the largest group (80 units) responded to all stimuli. Table 3 provides a summary of the number of responding and nonresponding units across the entire dataset, showing that 305 discriminated units, representing 75% of the total, responded to at least one of the stimuli. Table 3 also shows that relatively few units responded solely to the three general stimulus types (looming, translating or compound) or movement constrained to either the frontal or rear visual field. However, 49 units responded only to a combination of looming and compound motion, that is, no response to pure translation. Figure S1 shows matrices of responding units across the entire dataset and stimulus type, including from the same locust (L16) represented in Figure 1C. In this example, a range of 10–13 units showed some response to any stimulus, eight responded to all stimuli and two (units 5 and 11) did not respond to pure translational motion. While unit 2 responded preferentially to motion within the anterior visual field, no units responded solely to motion within the posterior visual field (Table 3). Comparing across stimulus types, we found no significant differences in the median number (*H*_12_ = 20, *P *=* *0.06) or mean percent (*F*_12_ = 1.5, *P *=* *0.12) of units responding to each stimulus (Fig. 2C). These findings suggest that, generally, the pool of motion‐sensitive neurons was of similar size across different stimulus types. However, while there were no differences in the median number of responding units, the total number varied across stimulus types (Table 4) and not all units responded to each stimulus (Fig. S1).

**Table 3:** Unit response summary

	Number	%	Median (range)
Total units	405		20 (10–30)
Responding	305	75	16 (7–22)
Nonresponding	100	25	5 (0–13)
Looming	4		
Translation	1		
Compound	13		
Looming + compound	49		
Anterior visual field	1		
Posterior visual field	0		

Lower 6 rows refer to responses to stimuli that only contain the specified motion. Looming (45, 90 and 135); translation (A and P) compound (A45, A90, A135, P45, P90, P135); anterior visual field (45, A45); posterior visual field (135, P135).

**Table 4:** Responding units and dimensionality reduction across stimuli

Stimulus	45	90	135	A	P	A45	A90	A135	P45	P90	P135
#Units responding	213	231	222	173	175	219	201	211	188	205	228
#Components (PCA)	94	77	94	126	122	103	110	115	108	103	109
#Trends (DFA)	8	10	9	7	6	10	5	5	5	7	10

**Figure 3:**
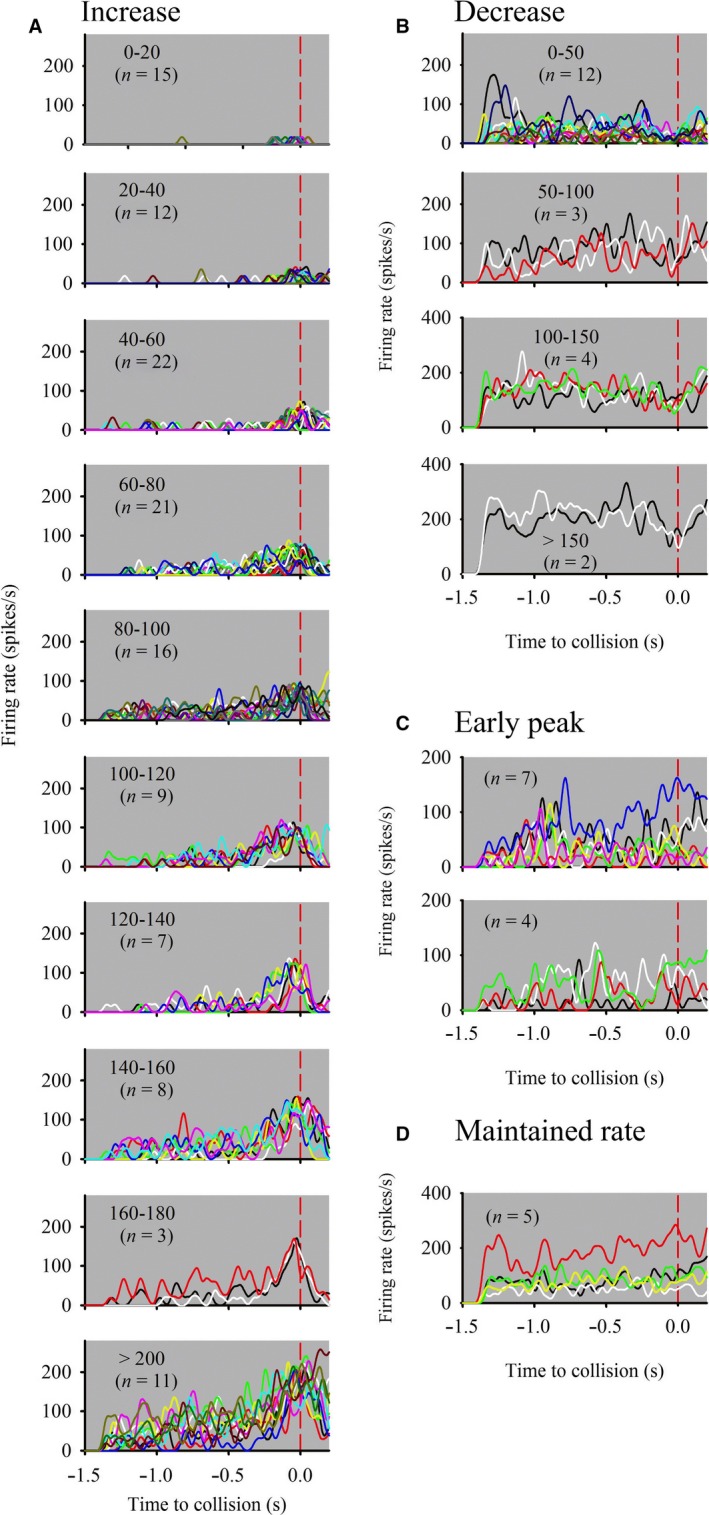
General response categories of units from all locusts responding to a direct loom from 90°. (A) Units that responded with an increasing firing rate near time of projected collision (*n* = 124). Based on relative distributions of peak firing rates, units were further subdivided into increasing rate bins of 20 spikes/sec (inset for each graph). No units showed peak firing rates in the range of 180–200 spikes/sec. (B) Units that responded with a decreasing firing rate near time of projected collision (*n* = 21). Based on relative distributions of the mean firing rate of the histogram, units were further subdivided into increasing rate bins of 50 spikes/sec (inset for each graph). (C) Units that responded with an early (top panel) or intermediate (bottom panel) peak firing (*n* = 11). (D) Units in which the firing rate increased early during the approach and maintained a relatively constant rate. For all graphs *n* = the number of units. Only 161 of 231 units that responded to a loom from 90° could be categorized (see text for details).

**Figure 4:**
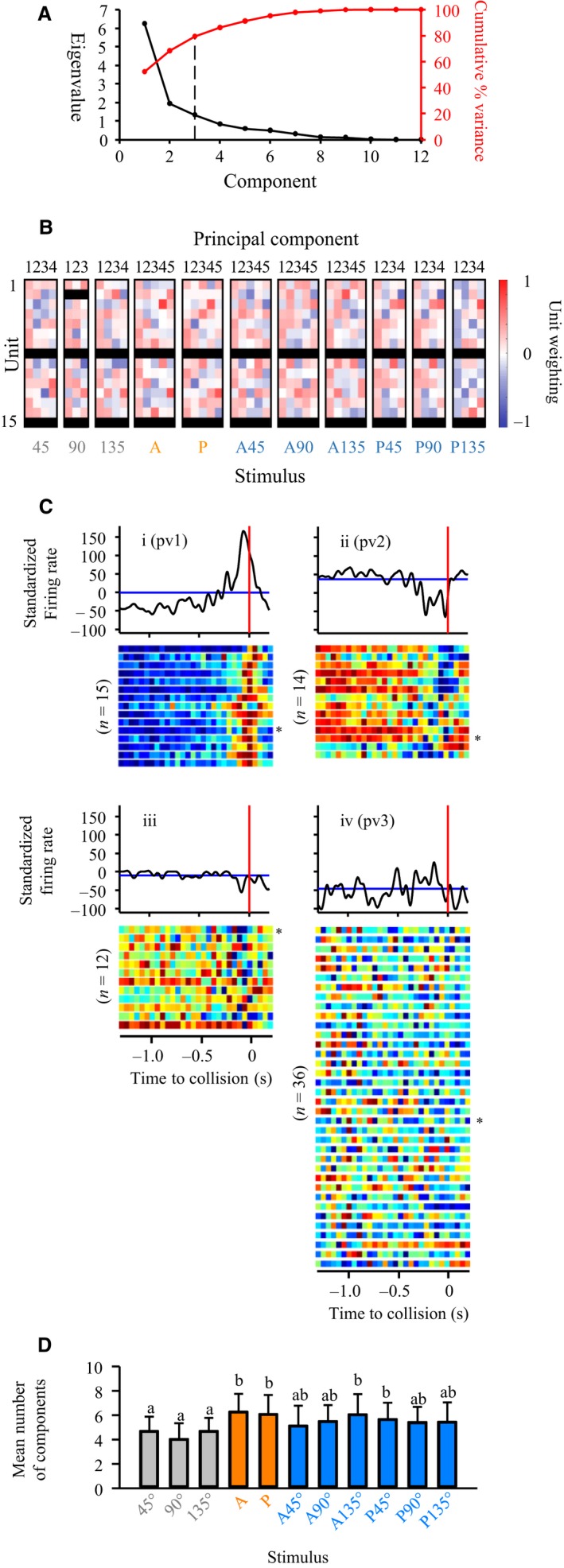
Principal component analysis of responding units revealed distinct population vectors. (A) Scree plot of data from a single locust (represented in Fig. 2A) showing eigenvalues (black line) and cumulative % variance (red line). The dashed vertical line shows the number of components (3) with eigenvalues >1 that explained at least 70% of the cumulative variance. These components were converted to population vectors and used for subsequent analysis. (B) Unit weighting within correlation matrices of components across all stimuli for the 15 units represented in Figs. 1C and 2A. Black cells are from units that did not respond to the stimulus. (C) Perievent time histograms of population vectors (pv) from locusts responding to a direct loom from 90°. The blue horizontal line represents a 95% confidence interval of the histogram and the red vertical line indicates the time of projected collision. pv1 (i), pv2 (ii), and pv3 (iv) represent corresponding components extracted from the same locust represented in A. A fourth component (iii) represents a component from a different locust. Heat maps below the histograms represent the standardized firing rate divided into 50 ms bins and color coded for similar population vector types across the entire dataset from all locusts such that deep blue is the lowest value and deep red is the highest value. Each row of the heat maps represents a single vector from one locust and the asterisks to the right indicate the heat map corresponding to the histogram. Numbers to the left of the heat maps represent the number of vectors within the vector type. See text for details. (D) The number of components that explained 70% of the variance in unit firing across all locust and stimulus types. Data represent the mean and positive standard deviation. Different letters above bars represent significant differences between stimuli. Significance assessed at *P *<* *0.05.

Unit responses to object motion across all stimulus types were highly variable across and within each stimulus type. As a first attempt to identify and classify patterns of unit response properties, we examined the 231 units from 20 locusts that responded to a disk looming from 90°. At this initial level of classification, we were able to classify four general response types from 161 units based on firing rate modulation during object approach (Fig. 3). These were as follows: (1) a firing rate increase near time of projected collision (Fig. 3A), (2) a firing rate decrease near time of projected collision (Fig. 3B), (3) an early to intermediate firing rate peak (Fig. 3C), and (4) an early firing rate increase that was maintained until time of projected collision (Fig. 3D). Those from category 1 were further subdivided into 10 subcategories based on peak firing rate increments of 20 spikes/s (Fig. 3A). Units from category 2 were further subdivided based on relative distributions of the mean firing rate of the histogram and placed into one of four increments of 50 spikes/s (Fig. 3B). Units from category three showed distinct peak firing rates earlier than those from category 1 and were distinguished by an early peak occurring from −1.0 to −0.75 sec before TOC or an intermediately timed peak that occurred 0.75 to 0.4 sec before TOC. There were relatively few units from category 4 and these could not be reliably subdivided further. While the remaining 70 units responded to a loom from 90°, the firing modulations were inconsistent and could not be classified. Therefore, it was not possible to classify all units based solely on common responses to looming.

Taken together variable tetrode positioning (see Materials and Methods) likely resulted in variability in recording the number of responding units across all stimuli, which limited our ability to unambiguously classify unit responses within a single stimulus type. Therefore, we carried out dimensionality reduction analyses to explore putative common patterns in responses within and across stimuli.

### Population vectors within individual locusts

Principal component analysis (see Materials and Methods) of units from one locust, represented in Figs. 1C and 2A, revealed three components that explained 70% of the cumulative variance and produced eigenvalues > 1 (Fig. 4A). From all sorted units in the same locust, we observed that the weighting of units in the major principal components differed across stimuli (Fig. 4B). After converting components from responses to a loom from 90° from all locusts (*n* = 20) into population vectors and plotting the PSTHs relative to TOC (Fig. 4C), we observed three distinct response types. The first (pv1, Fig. 4Ci) showed an increase in the firing rate near TOC, the second (pv2, Fig. 4Cii) showed a decrease near TOC and the third (pv3, Fig. 4Civ), showed no specific pattern. A population vector from another locust, showed a narrower valley in the firing rate near TOC (Fig. 4Ciii). While three response types (Fig. 4Ci, ii and iii) were reflective of the first two categories for individual units (Fig. 3A and B), they did not reveal subcategories nor an early or intermediate peak in the firing rate. However, the population categories were represented broadly across a pooled set of vectors following PCA of all units responding to a direct loom from 90°, as evidenced by the heat maps below the PSTHs in Figure 4C. The heat maps below pv3 (Figure 4Civ) show that many vectors responded with no categorical firing rate modulation. These findings suggest that while a subset of population vectors could be categorized based on firing rate modulation, another subset showed variable responses.

We also found that the average number of components varied between different stimulus types (Fig. 4D). Fewer vectors represented unit responses to direct looms (45, 90, and 135) compared to responses to pure translation starting in the anterior (A) or posterior (P) visual field or responses to compound trajectories with a longer duration translational component (A135, P45). This relationship between the stimulus type and average number of components contrasted that of the total number of components (Table 4). On average, PCA resulted in a dimensionality reduction of 48%, though the reduction was less pronounced for purely translating stimuli, suggesting that fewer components were required to represent responses to stimuli with a looming component.

These findings, firing rate variability in a subset of vectors responding to a direct loom from 90° and differences in the number of components across trajectories, suggest that categorization of general response properties remained ambiguous at this level of dimensionality reduction. Therefore, we performed a dynamic factor analysis (DFA) on the full set of components (see Materials and Methods).

### Common trends across population vectors

DFA revealed sets of common trends (CTs) among the PCA components pooled from 20 locusts for each stimulus type (see Materials and Methods for details). Table 2 demonstrates that in response to a loom from 90°, there were 10 trends in the standardized firing rates, based on the lowest value of Akaike's Information Criterion (AICc). The factor loadings of each of the 77 components (Table 4) in each of the 10 CTs for this stimulus are shown in Table S1.

**Figure 5:**
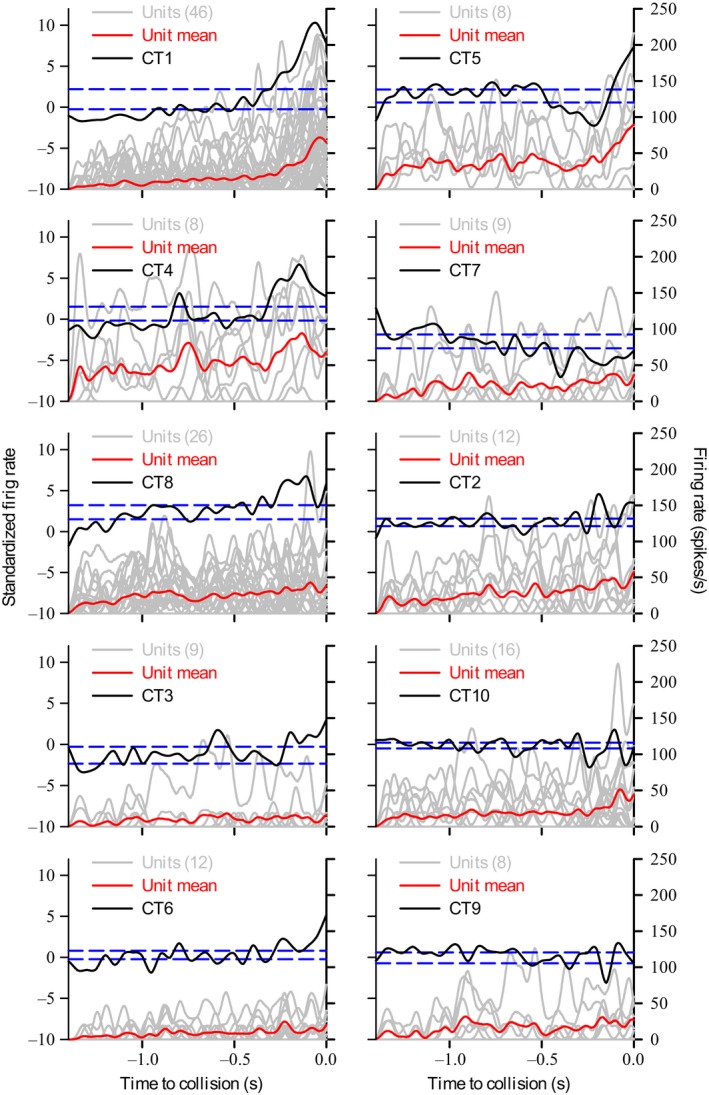
Dynamic factor analysis (DFA) of population vectors revealed 10 distinct trends in response to a disk looming from 90°. The analysis was performed on the 77 population vectors shown in Fig. 4C, representing 154 units across 20 locusts. The standardized firing rates (left graph axes) plotted as black lines and the 95% confidence interval of the CT histogram plotted as dashed blue lines. Firing rates (right axes) of units (gray lines) that contributed to each trend and the mean of the units (red lines) are overlaid. Numbers in parentheses are the number of units contributing to each trend (see Materials and Methods for details). Within the last 0.5 s of object approach (see Results), Trends 1, 4, 8, 3, and 6 (left panels) showed significant standardized firing rate increases, trends 5 and 7 (top right panels) showed significant decreases, trends 2, 10, and 9 (middle and lower right panels) showed sequences consisting of small peaks‐valleys‐peaks.

We then plotted the standardized firing rates of the CTs as PSTHs aligned to TOC in order to explore putative response types that coincide with individual unit or population vector responses. We characterized CT responses that extended outside of a 95% confidence limit of the histogram within 0.5 sec before collision, which is a behaviorally relevant time window (McMillan et al. 2013). Figure 5 shows CT responses (black lines) to a loom from 90°. As with units and vectors, we found a subset of trends that responded with an increased standardized firing rate near or leading up to TOC (Fig. 5 – left column of plots). A second subset, CT5 and 7, showed a firing rate decrease. CT2, CT10 and CT9 showed sequences of relatively small peak‐valley‐peak. Comparing to population vectors from PCA, CT1 resembled pv1(Fig. 4 Ci), CT5 resembled pv2 (Fig. 4Cii) and CT10 resembled vector responses from Figure 4Ciii. The 36 nonclassified responses from Figure 4Civ (e.g., pv3) may have contributed to CT2 and CT10. We also plotted the responses of units constituent to each CT (gray lines) and unit averages (red lines). The range of unit firing and differences between average firing and CTs suggest that trends are not a result of simple averaging of units and, instead, are result of weighted correlations resulting from dimensionality reduction.

### Composition and tuning of common trends

To examine the composition and responses of CTs across the stimulus types, we first determined the number of trends for each stimulus. Table 4 shows that trajectories with direct looming (45, 90, 135) or relatively brief translation (A45, P135) were represented by a greater number of trends (8–10), whereas purely translating (A, P) or translation to 90° or beyond (A90, A135, P45, P90) were represented by fewer trends (5–7). Thus, the number of trends were related to the nature of the stimulus, with fewer representing motion that is primarily translational.

**Figure 6:**
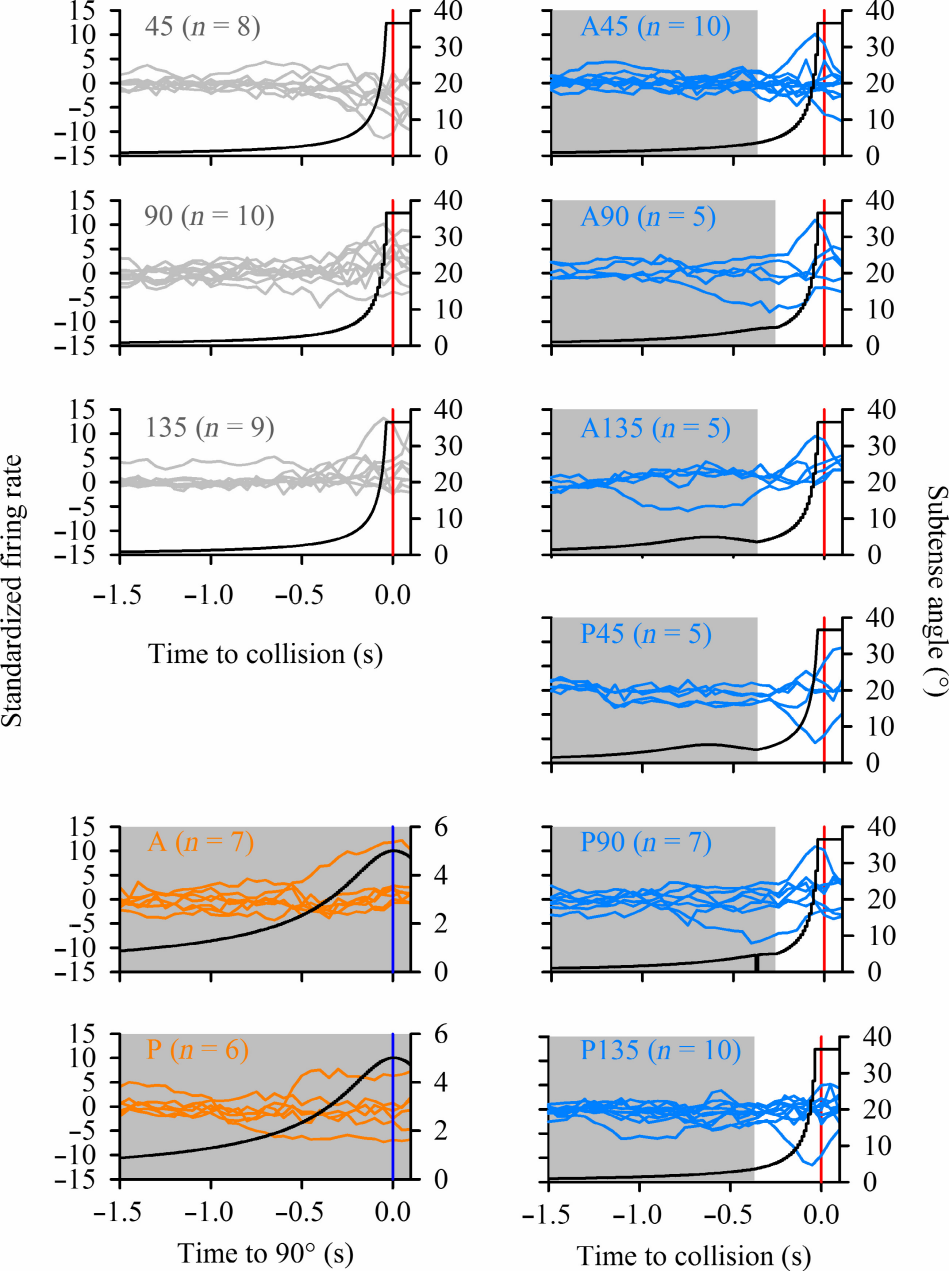
Overlay of DFA trend responses for each stimulus type. Gray lines indicate directly looming stimuli, orange indicates translating stimuli and blue indicates stimuli that transitioned from translating to looming. The left axes indicate the standardized firing rate. Insets indicate the trajectory type (*n* = number of trends). Black lines indicate the object subtense angle (right axes). Note the reduced scale for the subtense angle of translating stimuli (orange). For the *x*‐axis of looming and transitioning stimuli, 0 indicates time of projected collision (red vertical line) whereas for translating stimuli 0 indicates the time the object reached 90° azimuth (blue vertical line). Shaded areas represent durations of noncollision trajectories.

To examine how DFA common trend activity related to object motion, we plotted overlays of all CTs and object subtense angle (*θ*) versus TOC or T90 for each stimulus type (Fig. 6). For simple looms (45, 90, 135), the firing rate was relatively consistent across CTs prior to 0.5 sec before collision, though tuning was narrower for 45 and 135. Within 0.5 sec of collision, tuning broadened in response to all three simple looms, with concurrent increasing, decreasing and maintained firing rates. For translating trajectories (A and P), early tuning was relatively broad compared to simple looms and only one (A) or two (P) CTs showed increasing or decreasing firing rates between 0.5 and 1.0 sec before T90. Firing rates of the remaining CTs for each translating trajectory were maintained through to T90. For compound trajectories, (Fig. 6 right panels) CT firing rates were relatively consistent prior to the time of transition, except for one CT each in A90, A135, and P90, during which the standardized firing rate decreased 0.5–1.0 sec before transition. Within 0.5 sec of TOC or T90, or following a trajectory transition, CT firing broadened across a range of increasing or decreasing firing rates. Generally, these results demonstrate that correlated firing of units (in the form of common trends from DFA) dynamically tune across a broad range within individual stimulus types and that the tuning range varies with stimulus trajectories.

## Discussion

To the best of our knowledge, this is the first study to record parallel activity of multiple descending neurons from the nerve cord of locusts presented with simple and complex visual motion. This approach has allowed us to determine that correlated multineuronal activity relates to varying properties of behaviorally relevant visual stimuli. We discriminated 405 units (10–30 per locust) and classified them based on whether they responded to one of 11visual stimuli. Dimensionality reduction revealed population vectors within animals (PCA) and common trends of pooled population vectors (DFA). We found 5–10 common trends (CTs) that described general response properties depending on the duration of translational object motion across the locust visual field. Broadening of the CT relationship with the object size suggested distinct tuning across correlated neurons.

### Data recording and analysis

One of the limitations of multichannel, extracellular recording techniques is the inability to unambiguously identify individual neurons between animals due to subtle differences in tetrode positioning (Bhavsar and Heinrich 2015). While we took great care to position the tetrodes as accurately as possible in each preparation, we could not position the recording sites in the exact position relative to the same axons in each animal. Therefore, it was not possible to record, unambiguously, from the same neurons in each preparation. For this reason, we increased the sample size by pooling recordings across all animals for subsequent analysis. The caveat of positioning notwithstanding, many locust neurons can be characterized by responses to visual motion. Further, given that we discriminated 10–30 units per individual, it would be technically impossible to identify neurons using the gold standard of simultaneous, intracellular recordings. Nevertheless, given that the connective between the subesophageal and prothoracic ganglion contains ~60 axons with diameters >5 *μ*m (Rowell and Dorey 1967) and that we recorded from a stationary locust, it is likely that we sampled from a large percentage of the descending sensory interneurons within the cord. Another caveat is that we presented each stimulus only once to each locust. Therefore, we were not able to describe average responses of identified units or populations within each locust. By repeating sequences of fewer stimuli in future experiments we will be able to address whether multiple units show stereotyped responses. Nevertheless, the intent of the experiments reported here was to examine responses of multiple units across a wide range of simple and complex stimuli that we used in previous experiments when recording from DCMD alone. The number of stimulus presentations (13) combined with the duration of recording stability (approximately 1 h) precluded repeated presentations of each stimulus to each locust. While average responses can describe coding properties of units and population vectors, in the natural environment, specific object motion with the locust's visual field would likely not occur in a repetitive, controlled sequence. Therefore, our experimental design is an attempt to emulate aspects of variable natural object motion.

### Units compared to identified visual interneurons

The unit responses we observed likely reflect responses of previously identified descending visually sensitive interneurons in locusts. Multimodal M and S interneurons (Catton 1980) respond to visual stimuli across small subtense angles and display weaker inhibitory responses (Catton 1982) and less adaptation (Catton 1988) than DCMD. This property could result in weaker firing rate modulation when presented with object motion, similar to what we observed for units represented in Figure 3D. We may also have recorded from Descending Ocellar Neurons (DONs) that convey visual information from the ocelli to the thoracic ganglia (Rowell and Pearson 1983). However, DON responses are tuned to wide field motion such as that generated by yaw, pitch, or roll during flight. Similarly, the DNC neuron (Griss and Rowell 1986) responds to wide field motion produced by a diving banked turn to the contralateral side (Rowell and Reichert 1986). The Descending Ipsilateral Movement Detector (DIMD, Burrows and Rowell 1973) and other attitude deviation detectors (Rowell and Reichert 1986) receive input from the ipsilateral visual field and are thus likely not represented in the responses we observed following contralateral eye stimulation. It is highly likely that we recorded activity of the DCMD given the firing rate modulation we observed in units with peak firing rates from 160 to > 200 spikes/s (Fig. 3A), which are consistent with previous studies using similar stimuli (McMillan and Gray 2012). It is also likely that LDCMD is represented in one of the types shown in Figure 3A (100–120) with a firing rate that increases approximately 0.5 sec before collision and peaks slightly higher than 100 spikes/s, which is consistent with LDCMD responses to a looming disk traveling at 1 m/sec (Gray et al. 2010). Following more precise characterization of units through sequential use of a more restricted set of stimuli (see above), future experiments could then incorporate a greater range of visual stimuli, including wide field, to determine if the parallel responses we describe include previously identified neurons.

### Object motion relates to the distribution of responding neurons

Within many systems, ensemble reconfiguration may allow a level of functional plasticity that can accommodate complex sensory environments and which may be expressed to a greater or lesser extent across taxonomic groups. In the mouse primary visual cortex, coupling of individual neurons within a population varies but is independent of sensory preferences and may be more related to motor intention (Okun et al. 2015). In smaller, and more tractable nervous systems, however, it is likely that appropriate coding of variable sensory information requires neurons from a constrained pool to participate differentially in dynamic population activity. Multisensory neurons in the cockroach central complex respond to changes in antennal stimulation and light, and population responses are based on individual unit firing (Ritzmann et al. 2008). In the moth antennal lobe, odors are represented dynamically through restructuring of spatial and temporal components of ensemble responses (Daly et al. 2004). Here, we found that from a pool of 405 units, the absolute number that responded varied with the type of stimulus (Table 4 and Fig. S1), though the mean number and percent were consistent between individuals (Fig. 2C). These findings suggest that while the number of responding neurons is constrained, unit activity reflected in different response categories (Fig. 3) contribute to population vector activity in a context‐dependent manner (Fig. 4B).

### Composition of groups of correlated neurons varies with stimulus properties

Within individuals, spatiotemporal properties of dynamic stimuli activate suites of receptors in different patterns that are reflected in the composition and dimensionality reduction of activity in downstream neural ensembles. Dimensionality within the gustatory sensory cortex of rats grows linearly with ensemble size and predicts an upper bound proportional to pairwise correlations between neurons (Mazzucato et al. 2016), whereas in barrel cortex, emergent ensemble activity is invariant across large changes in stimulus amplitude (Jacobs et al. 2015). Coupling of neurons within populations of mouse visual cortex can be diverse, ranging from narrow to broad (Okun et al. 2015), demonstrating that dimensionality reduction can vary within a single sensory modality. Dimensionality reduction can also resolve tradeoffs between sensory resolution and processing requirements. Salamander retinal ganglion cells track motion using a population vector average that, though perhaps not globally optimal, can be implemented with computational efficiency within an ethologically relevant range of stimuli (Leonardo and Meister 2013). In rhesus monkey visual cortex, vector averaging revealed which neurons participated in population responses to changes in spatial properties of a visual stimulus (Priebe and Lisberger 2004). We found that, within individuals, the distribution (Fig. 4B) and, across all locusts, the number (Fig. 4D and Table 4) of components that explained at least 70% of the firing rate variability changed depending on the stimulus. We also distinguished population vectors based on three general types of responses to direct looms (Fig. 4C). Through DFA, we were able to further reduce the dimensionality of an ensemble pool from all individuals and observe responses to object motion from trends composed of populations vectors. This allowed us to disambiguate variability of units within individuals and identify common trends in responses to direct looms (Fig. 5) as well as their relationships to changes in object size during an approach (Fig. 6).

### Correlated neural responses across a range of spatiotemporal stimulus properties

Population responses of complex, dynamic sensory information can provide downstream neural circuits with computationally efficient decoding strategies to effect appropriate and flexible responses associated with behaviors. Population tuning curves of rabbit retinal ganglion cells are broad, minimizing large errors at the expense of higher average error and may be sufficient to encode a range of stimulus parameters including object velocity, size and angle of motion (Fiscella et al. 2015). Within rat barrel cortex, invariance of ensemble activity across large changes in stimulus amplitudes could be important for early stage sensory processing (Jacobs et al. 2015). Populations also encode across ranges of spatiotemporal stimulus properties. In *Drosophila* antennal lobe, populations of local inhibitory neurons encode stimulus intensity on multiple timescales with responses controlled by combinations of synaptic inputs and intrinsic properties (Nagel and Wilson 2016). Multiplexing in leech mechanosensory neurons encodes mechanosensory location and intensity via fast spiking touch sensory (T) cells and summed spike counts of pressure sensitive (P) cells, respectively (Pirschel and Kretzberg 2016). Gonzalez‐Bellido et al. (2013) found that 16 dragonfly visual neurons code a population vector that represents target direction across 360° and is provides reliable computation. Population responses can also be important for changing stimuli similar to what we tested here. Salamander retinal ganglion cells track motion using a population vector average that is efficient within an ethologically relevant range of stimuli (Leonardo and Meister 2013). Within the medial temporal area of the extrastriate visual cortex in Rhesus monkeys, population responses predict effects of spatial frequency and contrast on target speed estimations (Priebe and Lisberger 2004) and Macaque visual cortex multiplexes natural stimuli through coding on different time scales (Ayzenshtat et al. 2012). Consistent with our findings, neuronal populations in ferret visual cortex (V1) vary response properties (peak firing) when objects change direction (Wu et al. 2011).

In locusts, adaptive coding in a set of auditory sensory neurons can circumvent ambiguity created by adaptation and allow the system to temporally disambiguate auditory patterns and location (Hildebrandt et al. 2015). In the context of the locust visual system, since correlated firing of presynaptic inputs to the LGMD can provide selectivity to looming objects (Jones and Gabbiani 2010) it is reasonable to predict that population responses of descending interneurons could provide a computationally efficient way to convey important information to motor centers involved in generating avoidance responses. Without coordinated population responses, we may expect that the total firing of all individual units would provide a poor representation of objects traveling along complex trajectories. We found that low dimension correlated neural responses relate to spatiotemporal aspects within a single stimulus type (Fig. 4) and that the relationships change depending on the complexity of object motion **(**Fig. 6). Future experiments that incorporate multichannel recordings from tethered locusts “flying” in a closed‐loop arena will allow us to directly address whether correlated activity converges to provide an efficient neural code for downstream motor enters that drive flight steering behavior.

The findings we present here directly address a long‐standing requirement to understand fundamental properties of correlated neural firing to more accurately describe the make‐up, responses, and coding properties of neural populations that are implicated in controlling a complex behavior. Building on our data, it will now be possible to identify populations and their responses to a wide range of visual stimuli ranging from local to wide field motion to more accurately describe the tuning of units and populations and clearly determine if range fractionation is embedded within population responses, as suggested by Figure 6. We will also be able to include concomitant intracellular recording techniques to investigate presynaptic visual inputs from the optic lobes to the populations as well as postsynaptic outputs of circuits within the thoracic ganglia that control motor centers associated with avoidance behaviors. The latter will allow us to determine if and how populations converge onto motor elements important for escape behaviors. For example, while DCMD makes relatively weak connections onto subsets of flight motorneurons (Simmons 1980) it is not known if other motion‐sensitive interneurons converge and summate to produce stronger excitation of flight motorneurons, which could account for variable responses to approaching objects during flight.

## Conflict of Interest

No conflicts of interest, financial or otherwise, are declared by the author(s).

## Data Accessibility

## Supporting information




**Figure S1.** Response matrices of the entire data set of 405 units from 20 locusts. For each locust (L), the top and bottom matrices represent units recorded from tetrode 1 and 2, respectively. Filled and open cells identify responding and non‐responding units, respectively. Numbers in parentheses represent the number of units responding to any of the stimuli. Units are represented in rows and the stimulus is represented in columns. Grey stimulus numbers and cells indicate directly looming stimuli, orange indicates translating stimuli and blue indicates stimuli that transition from translating to looming. The legend identifies the specific stimulus (see Materials and Methods for details).
**Table S1.** Factor loadings from the dynamic factor model for a 90° loom (Supp. Table 2) including ten common trends (CT1 –CT10) and an unequal covariance structure.
