# Supplementary material for: Complex object motion represented by context‐dependent correlated activity of visual interneurones
Source: Physiol Rep. 2017 Jul 17;5(14):e13355. doi: 10.14814/phy2.13355 (PMC5532489; doi:10.14814/phy2.13355)
Supplement: Supplementary file 1 — Figure S1. Response matrices of the entire data set of 405 units from 20 locusts. For each locust (L), the top and bottom matrices represent units recorded from tetrode 1 and 2, respectively. Filled and open cells identify responding and non‐responding units, respectively. Numbers in parentheses represent the number of units responding to any of the stimuli. Units are represented in rows and the stimulus is represented in columns. Grey stimulus numbers and cells indicate directly looming stimuli, orange indicates translating stimuli and blue indicates stimuli that transition from translating to looming. The legend identifies the specific stimulus (see Materials and Methods for details). Table S1. Factor loadings from the dynamic factor model for a 90° loom (Supp. Table 2) including ten common trends (CT1 –CT10) and an unequal covariance structure. [file PHY2-5-e13355-s001.pdf]

Supporting Figure 1

Response matrices of the entire data set of 405 units from 20 locusts. For each locust (L), the top and bottom matrices represent units recorded from tetrode 1 and 2, respectively. Filled and open cells identify responding and non-responding units, respectively. Numbers in parentheses represent the number of units responding to any of the stimuli. Units are represented in rows and the stimulus is represented in columns. Grey stimulus numbers and cells indicate directly looming stimuli, orange indicates translating stimuli and blue indicates stimuli that transition from translating to looming. The legend identifies the specific stimulus (see Materials and Methods for details).

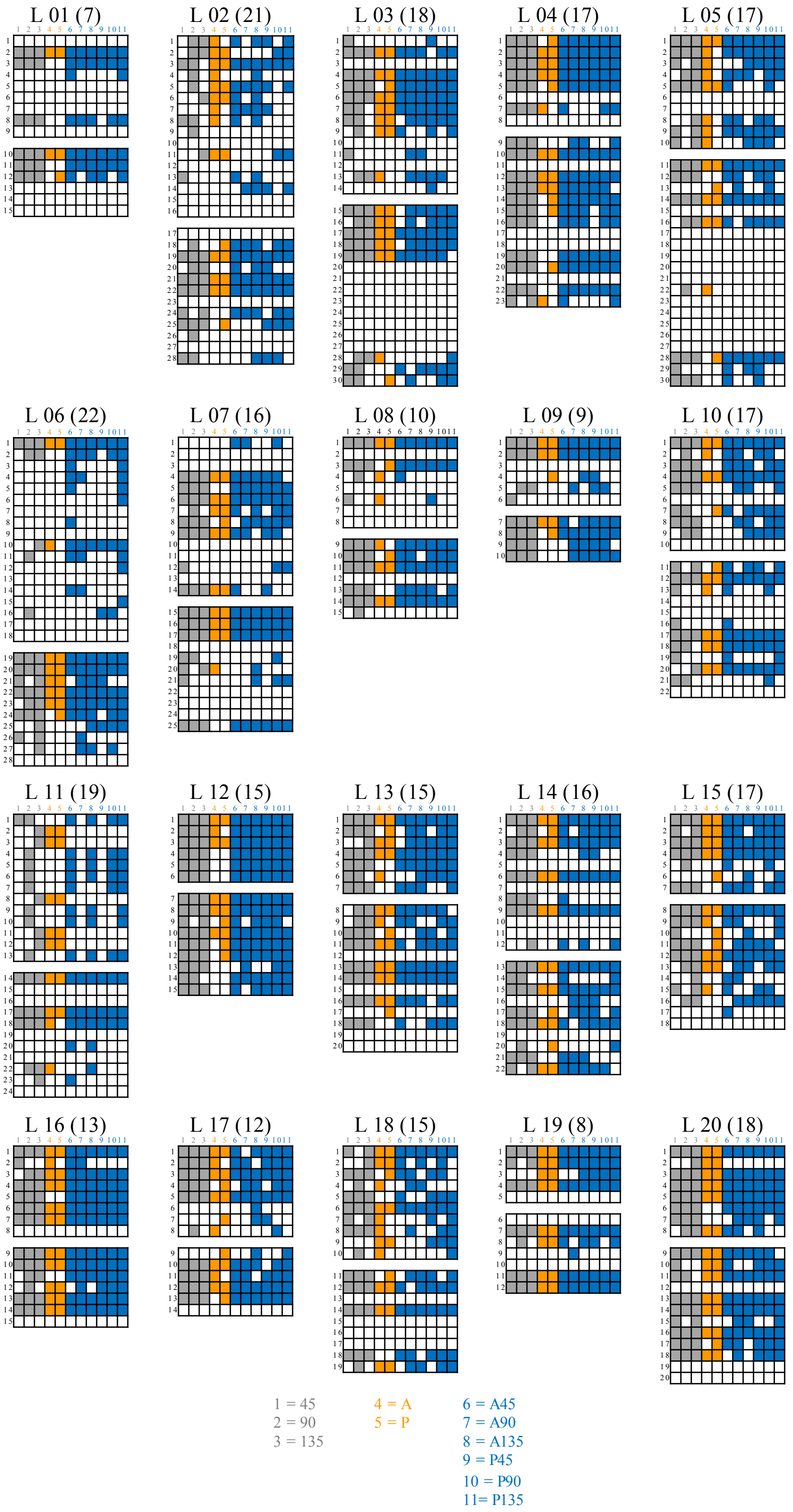
L 01 (7)
L 02 (21)
L 03 (18)
L 04 (17)
L 05 (17)
L 06 (22)
L 07 (16)
L 08 (10)
L 09 (9)
L 10 (17)
L 11 (19)
L 12 (15)
L 13 (15)
L 14 (16)
L 15 (17)
L 16 (13)
L 17 (12)
L 18 (15)
L 19 (8)
L 20 (18)
1 = 45
2 = 90
3 = 135
4 = A
5 = P
6 = A45
7 = A90
8 = A135
9 = P45
10 = P90
11= P135

Supporting Table 1

Factor loadings from the dynamic factor model for a 90° loom (Supp. Table 2) including ten common trends (CT1 – CT10) and an unequal covariance structure.

	Unit	CT1	CT2	CT3	CT4	CT5	CT6	CT7	CT8	CT9	CT10
1.	L01_Stim09_01	0.32	0.26	0.09	-0.33	0.17	0.28	0.00	-0.15	-0.13	-0.17
2.	L01_Stim09_02	-0.08	0.53	-0.31	-0.08	-0.12	0.09	0.07	-0.29	-0.41	-0.72
3.	L02_Stim09_01	-0.22	-0.03	-0.31	0.04	-0.07	-0.04	0.09	0.02	-0.01	-0.05
4.	L02_Stim09_02	0.14	0.03	0.35	-0.04	0.01	-0.01	-0.05	0.03	0.03	0.08
5.	L02_Stim09_03	0.12	0.11	-0.50	0.11	0.08	0.14	-0.14	-0.21	-0.14	-0.24
6.	L02_Stim09_04	-0.02	-0.02	0.56	-0.05	0.00	-0.11	0.09	0.03	0.00	-0.04
7.	L02_Stim09_05	0.08	0.30	0.15	-0.09	-0.12	0.26	-0.20	-0.15	0.17	0.04
8.	L02_Stim09_06	0.11	-0.07	-0.59	0.01	0.06	-0.27	0.11	0.17	-0.25	-0.09
9.	L03_Stim09_01	0.48	0.13	0.11	-0.16	-0.01	0.12	-0.03	-0.21	0.12	-0.01
10.	L03_Stim09_02	-0.11	-0.44	0.12	0.08	0.13	-0.11	0.08	0.39	0.24	0.06
11.	L03_Stim09_03	-0.05	-0.63	-0.02	-0.03	0.05	0.03	0.03	0.09	0.01	0.15
12.	L03_Stim09_04	-0.13	-0.06	-0.19	0.07	0.16	0.06	0.21	0.42	-0.09	0.11
13.	L04_Stim09_01	0.35	-0.10	0.01	0.01	0.03	-0.03	0.00	0.05	0.18	0.12
14.	L04_Stim09_02	0.22	-0.20	0.03	-0.27	-0.03	0.05	0.36	0.30	1.06	0.10
15.	L04_Stim09_03	-0.18	-0.46	-0.01	0.21	-0.09	0.08	-0.12	0.41	-0.03	0.35
16.	L04_Stim09_04	0.07	0.21	0.08	-0.19	0.09	0.03	0.11	0.11	-0.16	-0.50
17.	L05_Stim09_01	0.33	-0.01	-0.03	-0.08	0.00	-0.04	-0.19	-0.10	0.13	-0.15
18.	L05_Stim09_02	-0.34	0.42	0.02	-0.13	-0.13	0.66	-0.18	-0.27	0.15	-0.53
19.	L05_Stim09_03	0.18	0.04	-0.07	-0.03	-0.11	-0.71	0.37	0.40	0.42	-0.12
20.	L05_Stim09_04	-0.22	0.31	0.02	-0.06	-0.04	0.03	0.20	0.10	-0.76	0.00
21.	L06_Stim09_01	-0.62	0.07	-0.04	0.50	0.05	0.11	-0.18	-0.21	-0.02	0.20
22.	L06_Stim09_02	0.01	-0.12	0.14	-0.07	-0.24	-0.24	0.18	0.64	-0.07	-0.06
23.	L06_Stim09_03	0.24	0.03	0.15	-0.15	-0.03	-0.01	-0.26	-0.24	-0.39	-0.17
24.	L06_Stim09_04	0.14	-0.05	0.05	-0.18	0.11	-0.05	0.15	0.43	-0.04	0.64
25.	L06_Stim09_05	0.29	-0.45	-0.21	-0.14	-0.06	0.01	0.17	0.02	-0.08	-0.32
26.	L06_Stim09_06	-0.03	0.13	0.21	-0.43	-0.03	-0.10	-0.09	0.24	0.11	-0.08
27.	L06_Stim09_07	0.03	-0.11	-0.03	-0.08	-0.03	0.02	0.03	0.39	0.11	-0.03
28.	L07_Stim09_01	-0.35	0.06	0.01	0.00	0.03	-0.04	-0.02	-0.09	-0.24	-0.21
29.	L07_Stim09_02	0.24	-0.56	-0.15	-0.33	-0.26	0.12	-0.12	0.08	0.22	-0.55
30.	L07_Stim09_03	0.11	0.03	-0.32	0.04	0.23	0.17	-0.12	-0.27	-0.84	-0.30

31.	L07_Stim09_04	-0.03	0.51	-0.03	0.13	0.07	0.03	0.00	-0.03	-0.21	0.03
32.	L08_Stim09_01	-0.15	-0.02	0.02	0.00	-0.06	0.19	0.17	-0.02	-0.03	-0.03
33.	L08_Stim09_02	-0.07	-0.29	-0.14	0.03	-0.05	0.74	0.33	0.14	-0.12	0.16
34.	L08_Stim09_03	-0.04	-0.43	-0.26	-0.14	-0.11	0.33	0.29	0.32	-0.50	-0.09
35.	L08_Stim09_04	0.00	-0.47	0.04	0.09	0.01	0.03	0.11	0.01	-0.02	0.11
36.	L09_Stim09_01	0.26	-0.07	0.05	0.06	0.21	0.01	-0.01	-0.12	0.14	-0.15
37.	L10_Stim09_01	0.19	0.06	-0.01	0.09	0.13	-0.06	0.04	0.07	-0.05	0.16
38.	L10_Stim09_02	-0.06	0.10	-0.02	0.30	0.10	-0.15	0.10	0.21	-0.25	0.03
39.	L10_Stim09_03	0.02	-0.79	-0.42	-0.14	-0.02	0.40	0.01	0.35	0.08	-0.21
40.	L10_Stim09_04	-0.12	0.26	-0.24	0.24	0.30	-0.03	0.37	0.09	-0.52	-0.31
41.	L11_Stim09_01	-0.35	-0.02	-0.09	-0.18	-0.13	0.07	-0.14	0.09	0.11	-0.20
42.	L11_Stim09_02	-0.03	0.12	-0.08	-0.21	0.05	-0.02	-0.27	-0.03	-0.24	-0.39
43.	L11_Stim09_03	0.06	-0.07	-0.01	-0.05	0.04	0.00	0.31	0.02	-0.03	0.11
44.	L11_Stim09_04	-0.21	0.01	0.10	0.48	-0.02	0.00	-0.19	0.00	-0.05	0.13
45.	L12_Stim09_01	0.19	0.04	0.17	0.00	0.00	-0.02	-0.09	0.05	-0.02	-0.10
46.	L12_Stim09_02	-0.11	-0.15	-0.06	0.00	0.02	0.09	0.13	0.08	0.12	0.65
47.	L12_Stim09_03	0.00	-0.32	-0.32	0.12	0.31	0.07	0.24	-0.13	0.23	0.00
48.	L12_Stim09_04	0.03	-0.73	-0.53	-0.07	0.10	0.67	-0.17	-0.15	-0.11	-0.21
49.	L13_Stim09_01	-0.51	-0.22	0.15	0.22	0.04	-0.15	-0.12	0.04	-0.02	0.26
50.	L13_Stim09_02	-0.13	0.55	0.09	0.25	-0.05	0.01	-0.03	-0.53	-0.08	-0.37
51.	L13_Stim09_03	-0.19	0.34	-0.15	0.40	-0.11	0.13	0.19	0.03	-0.23	-0.44
52.	L13_Stim09_04	0.06	-0.05	0.19	-0.60	-0.11	0.25	-0.01	0.18	0.08	-0.08
53.	L13_Stim09_05	-0.02	0.04	0.08	0.14	-0.03	-0.10	-0.52	-0.29	0.04	0.17
54.	L15_Stim09_01	0.30	-0.06	-0.17	-0.01	0.07	0.09	0.03	0.07	-0.04	-0.06
55.	L15_Stim09_02	0.04	0.27	-0.05	-0.19	-0.02	-0.12	0.22	-0.05	-0.12	-0.14
56.	L15_Stim09_03	0.06	0.08	0.14	-0.18	-0.11	0.24	0.16	0.51	0.11	0.31
57.	L15_Stim09_04	-0.42	0.52	0.68	-0.13	-0.10	0.09	-0.34	-0.21	0.53	-0.15
58.	L15_Stim09_05	0.00	-0.39	-0.17	0.28	0.20	0.03	-0.07	-0.03	-0.15	-0.04
59.	L16_Stim09_01	0.56	-0.49	-0.05	-0.11	-0.03	0.07	0.08	0.04	0.18	0.04
60.	L16_Stim09_02	-0.44	0.25	0.24	-0.02	0.19	0.16	0.02	0.27	0.11	0.82
61.	L16_Stim09_03	0.10	-0.16	-0.09	-0.09	-0.15	-0.13	0.42	0.44	-0.61	-0.07
62.	L17_Stim09_01	0.39	-0.02	0.21	-0.11	-0.14	0.05	0.01	-0.16	-0.08	0.07
63.	L17_Stim09_02	-0.04	-0.13	-0.01	0.03	0.03	0.26	0.05	-0.12	-0.80	0.05
64.	L17_Stim09_03	0.06	0.21	0.00	-0.02	-0.17	0.08	0.26	0.09	-0.01	-0.28
65.	L17_Stim09_04	0.01	-0.06	-0.28	0.00	0.07	-0.03	-0.05	0.14	0.24	-0.08
66.	L18_Stim09_01	0.24	0.20	0.00	-0.02	0.03	-0.11	0.03	0.06	0.07	-0.23

67.	L18_Stim09_02	0.07	-0.04	-0.07	0.06	0.34	-0.05	-0.05	-0.05	-0.16	0.09
68.	L18_Stim09_03	-0.14	-0.04	0.16	0.08	0.04	0.04	-0.06	0.10	-0.42	0.49
69.	L18_Stim09_04	-0.09	0.21	0.26	0.24	-0.05	-0.54	-0.09	-0.29	0.32	-0.04
70.	L18_Stim09_05	-0.01	0.09	0.04	0.39	0.30	0.00	-0.01	-0.24	0.07	0.50
71.	L19_Stim09_01	0.38	-0.01	0.05	-0.08	-0.08	-0.10	-0.04	0.08	-0.06	-0.04
72.	L19_Stim09_02	0.06	0.00	-0.07	-0.05	0.08	0.02	-0.29	-0.09	-0.13	0.01
73.	L19_Stim09_03	0.07	-0.03	-0.21	-0.12	0.16	0.06	-0.09	0.04	0.01	-0.06
74.	L20_Stim09_01	0.33	0.01	-0.04	0.04	0.11	-0.14	-0.02	-0.04	-0.03	-0.03
75.	L20_Stim09_02	-0.02	0.05	-0.07	0.21	0.07	-0.01	-0.06	0.07	-0.39	0.00
76.	L20_Stim09_03	0.19	0.07	0.15	0.05	-0.07	-0.19	-0.04	-0.11	-0.33	0.70
77.	L20_Stim09_04	-0.02	-0.11	-0.20	-0.26	0.23	-0.13	0.17	0.06	-0.14	-0.58
